# Efficacy of Pleuran (*β*-Glucan from *Pleurotus ostreatus*) in the Management of Herpes Simplex Virus Type 1 Infection

**DOI:** 10.1155/2020/8562309

**Published:** 2020-04-13

**Authors:** Ingrid Urbancikova, Dana Hudackova, Juraj Majtan, Zuzana Rennerova, Peter Banovcin, Milos Jesenak

**Affiliations:** ^1^Department of Pediatrics, P. J. Safarik University, Faculty of Medicine, Children Faculty Hospital, Trieda SNP 1, 040 11 Kosice, Slovakia; ^2^Department of Pediatric Infectology, Children Faculty Hospital, Faculty of Medicine, Trieda SNP 1, 040 11 Kosice, Slovakia; ^3^Institute of Molecular Biology, Slovak Academy of Sciences, Dubravska Cesta 21, 845 51 Bratislava, Slovakia; ^4^Pneumo-Allergo Centre Ltd., Udernicka 1, 851 01 Bratislava, Slovakia; ^5^Department of Pediatric Pulmonology and Phthisiology, Faculty of Medicine, Slovak Medical University, National Institute of Children Diseases, Krajinska 91, 825 56 Bratislava, Slovakia; ^6^Department of Paediatrics, Jessenius Faculty of Medicine, Comenius University in Bratislava, Kollarova 2, 036 59 Martin, Slovakia; ^7^Department of Pulmonology and Phthisiology, Jessenius Faculty of Medicine, Comenius University in Bratislava, Kollarova 2, 036 59 Martin, Slovakia; ^8^Department of Clinical Immunology and Allergology, Comenius University in Bratislava, Kollarova 2, 036 59 Martin, Slovakia

## Abstract

One of the highly prevalent viral pathogens among children and adults causing infection, clinically presenting as herpes labialis, is herpes simplex virus type 1 (HSV-1). The long-term administration of acyclovir, a standard regimen for therapy against HSV-1 infections, can cause viral resistance against this drug. Therefore, the development of natural drugs with low toxicity that are able to enhance host antiviral defense against HSV infection is needed. *β*-Glucans represent a type of biologically active molecules possessing antiviral properties. The goal of this study was to investigate the clinical and immunomodulatory effect of *β*-glucan pleuran (insoluble *β*-1,3/1,6-D-glucan isolated from *Pleurotus ostreatus*) based supplements on the duration and intensity of herpes symptoms and on the incidence rate and duration of acute respiratory symptoms and intercurrent diseases in HSV-1 positive patients. Ninety patients were randomised into active and placebo groups. Active treatment with pleuran in systemic application caused a significantly shorter duration of herpes simplex symptoms compared to the placebo group. During the preventive phase (120 days), the duration and severity of respiratory symptoms were lower in the active group compared to the placebo group; however, a significant difference was found only in the case of cough. No significant side effects were observed during both phases of the clinical trial (acute and preventive). Obtained results suggest that the use of pleuran seems to be a promising approach in the treatment of acute HSV-1 with beneficial effect on the respiratory tract symptoms and infections.

## 1. Introduction

*β*-Glucans are glucose polymers derived from a variety of sources, including yeast, grain, alga, and fungus, and belong to the class of drugs known as biological response modifiers [1]. Nowadays, *β*-glucans have attracted considerable attention due to their anti-infective, antiviral, antitumor, and immunomodulatory activities as well as wound healing properties [2–7]. As for their antiviral effect, *β*-glucans have shown excellent activity against a broad spectrum of plant, animal, and human viruses, such as the infectious haematopoietic necrosis virus [8], spring viremia of carp virus [9], dengue virus [10], herpes simplex virus type 1 (HSV-1) [11], human immunodeficiency virus 1 [12], influenza virus [13, 14], and tobacco mosaic virus [15]. Despite *in vitro* and *in vivo* evidence of the antiviral effect of *β*-glucans, to date, only a few human and animal clinical studies have been conducted utilizing *β*-glucans in treatment or prevention of viral infections [16–20].

The antiviral effect of *β*-glucans can be mediated directly through the inhibition and/or disruption of virus particles or indirectly by enhancing host antiviral defense. The mechanism of *β*-glucan's action is mediated through several receptors, especially the dectin-1 receptor, toll-like receptors (TLR 2, 4, and 6), complement receptor 3 (CR3), scavenger receptor, and lactosylceramide [21]. The most important is the dectin-1 receptor, which is highly expressed in many immunocompetent cells, such as dendritic cells (DC), neutrophils, eosinophils, macrophages, monocytes, and several T lymphocytes [22, 23].

One of the highly prevalent viral pathogens among children and adults causing infection, clinically presenting as herpes labialis, is herpes simplex virus type 1 (HSV-1). Acyclovir, a nucleoside analogue, has been used as a standard regimen for the control of HSV-1 by interfering with viral DNA synthesis. However, the prolonged use of acyclovir, in particular in immunocompromised patients, has led to viral resistance against this drug. Acyclovir-resistant HSV strains have been isolated in 5–10% of patients and are associated with significant morbidity [24]. Furthermore, acyclovir has low bioavailability and possesses a wide array of side effects, which can limit its short- and long-term use in children. Therefore, current research is focused on the development of natural drugs that are able to enhance host antiviral defense against HSV-1 infection.

Fungal and oat *β*-glucans exhibit *in vitro* antiherpetic activity and inhibit viral adsorption, penetration, and cell-to-cell spread [11, 25, 26] and increase the macrophage proinflammatory cytokine response to HSV-1 infection [27]. Therefore, they may represent potential prophylactic/treatment agents in HSV-1 infection and can decrease susceptibility to viral infection following oral administration of *β*-glucans.

The aim of the present study was to investigate the clinical and immunomodulatory effect of *β*-glucan pleuran (insoluble *β*-1,3/1,6-D-glucan isolated from *Pleurotus ostreatus*) based supplements on the duration and intensity of herpes symptoms (10-day acute treatment phase of HSV-1 infection) and on the incidence rate and duration of acute respiratory symptoms and intercurrent diseases in HSV-1 positive patients (120-day preventive phase).

## 2. Materials and Methods

### 2.1. Subjects

A multicentre, randomised, double-blind, placebo-controlled study was conducted by the Department of Infectious Diseases, Children Faculty Hospital in Kosice and 6 outpatient paediatric clinics across Slovakia between November 2014 and December 2017.

The study population included 90 patients aged over 6 years with herpes simplex facialis/labialis in one of the first three stages (prodromal/tingle stage, blister stage, or weeping stage) at the time of the screening visit.

Subjects with a history of severe primary or secondary immunodeficiency, invasive bacterial infection (e.g., sepsis and meningitis) in the prior 14 days and product allergies or intolerance as well as those who had used some systemic antiviral treatment (acyclovir, valacyclovir, or famciclovir) or any immunomodulatory therapy (bacterial lysates, *β*-glucans, probiotics, prebiotics, *Echinacea*, enzyme therapy, inosine pranobex, azoximer bromide, etc.) in the prior 28 days or those who were pregnant, breastfeeding, and unable to complete diary cards were excluded from the study.

The patients or their parents/tutors were informed about the conduction of the study, and their written informed consent was obtained.

### 2.2. Study Design

The study consisted of the following 2 phases: acute treatment phase (the first 10 days) and preventive phase (120 days after herpes lesion treatment). Subjects that met all the inclusion criteria were randomised into two different treatment groups to receive either *β*-glucan-based supplement or placebo. During the acute treatment phase, subjects in the active group (*n* = 49) were given 1 capsule of combined supplement containing pleuran 300 mg, vitamin C 160 mg, and zinc 10 mg (Imunoglukan P4H® ACUTE!) every morning on an empty stomach for 10 days as well as the recommended antiherpetic treatment (topical antiviral agents, analgesics, wound healing creams, or herpes patches). The placebo group (*n* = 41) followed the same treatment schedule including antiherpetic products but was given 1 capsule containing only 160 mg of vitamin C and 10 mg of zinc. Randomization was performed at the beginning of the study, and the unblinding was done after the completion of all the data. Active treatment and placebo did not differ in the shape, colour, taste, or smell.

A total of 77 out of 90 patients were followed up during the subsequent 120-day preventive phase. Subjects from the active and placebo groups were required to take 1 capsule of combined supplement containing 100 mg of pleuran and 100 mg of vitamin C (Imunoglukan P4H®) and placebo (100 mg of vitamin C), respectively, every morning on an empty stomach.

The active substance of the administered natural products was isolated by unique and patented technology from *Pleurotus ostreatus*. It was previously identified and chemically characterized by Karacsonyi and Kuniak [28].

The study protocol followed the guidelines of the Declaration of Helsinki and was approved by the Ethics Committees of the Children's Faculty Hospital in Kosice and of the Kosice Self-Governing Region.

Patients underwent five clinical visits during the whole study period: V0 (day 0)—screening visit on recruitment, V1 (day 10)—after 10 days of acute treatment, V2 (day 40)—after 30 days of preventive treatment, V3 (day 70)—after 60 days of preventive treatment, and V4 (day 130–the end of the study)—after 120 days of preventive treatment. At the time of inclusion, the anthropometric data and data on herpes occurrences in the last 6 and 12 months before enrolment as well as data on the stage, location, size of herpes efflorescence, and therapy applied were documented in all subjects.

During the acute treatment phase, study subjects maintained daily diary cards on which they noted the appearance, duration, and intensity of herpes symptoms (itching, redness, swelling, pain, exudation, scab, haemorrhage, and negative impact on daily activities) on a visual analogue scale from 0 (no symptoms) to 10 (the highest intensity). On days 0, 5, and 10, they measured the size of herpes efflorescence. At the end of the acute treatment phase, the opinions of patients or their parents/tutors about the treatment effect (0—no effect; 1—good; 2—very good) were recorded by a physician.

During the 120-day preventive phase, the following data were evaluated based on the diary cards of patients: the incidence of acute respiratory symptoms (rhinitis, cough, sore throat, earache, and fever) and intercurrent diseases (abdominal pain, aphthous stomatitis, labial herpes, shingles, skin rash, and mycotic infections), their intensity on scale 0 (no symptoms) to 3 (the highest intensity) and their duration (in days), and use of antibiotics and other concomitant therapy.

During the whole study, the tolerability of the studied products and the incidence of adverse events were also recorded.

### 2.3. Statistical Analysis

Statistical analysis was performed using the Statistical Package for the Social Sciences (SPSS) version 23.0 for Windows. Continuous variables were expressed as mean values with standard error means (SEMs), and the nominal variables were expressed as numbers and percentages. Analyses of the data included testing the differences in prevalence and comparison of the means by using the chi-square test (or Fisher's exact test, where appropriate) and Student's *t*-tests in cases of continuous data. In all cases, a *P* value less than 0.05 was considered statistically significant.

## 3. Results

A total of 90 patients were enrolled into the study, of which 87 (96.7%) completed the acute treatment phase and 77 subjects (85.6%) were followed up during the preventive phase. Three patients from the active group dropped out from the study during the acute treatment phase (diarrhoea after the first dose, nausea, and noncompliance with the study protocol). Ten patients (7 in the active group and 3 in the placebo group) decided to withdraw from the study prematurely after the acute treatment phase due to personal reasons and noncompliance with the study protocol. Demographic characteristics are summarized in Table 1.

**Figure 1:**
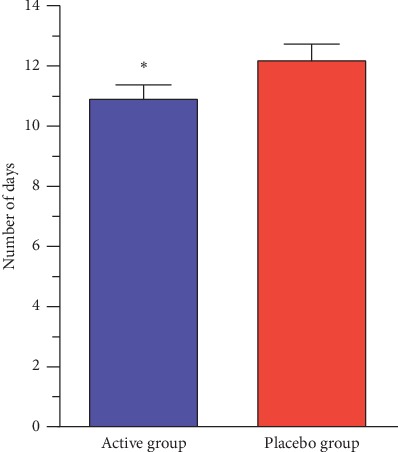
Duration of herpes symptoms in active and placebo groups during the acute treatment phase. ^*∗*^Significant difference (*P* < 0.05) in the number of days compared to the placebo group. Data represent mean ± SEM.

In the active group, a higher proportion of females [32 (65.3%) vs. 21 (51.2%); *P*=0.176] and a higher average age (25.3 ± 2.3 years vs. 17.4 ± 1.5 years; *P*=0.005) were found. No significant differences regarding the number of patients in different stages of herpes infections (prodromal/tingle stage, blister stage, or weeping stage) or the number of herpes exacerbations during the last 6 to 12 months before the study were observed. The patients from the active group had a significantly shorter duration of herpes symptoms leading to their earlier improvement compared to the patients treated with placebo (11.0 ± 0.4 days vs. 12.2 ± 0.5 days; *P*=0.046) (Figure 1).

**Figure 2:**
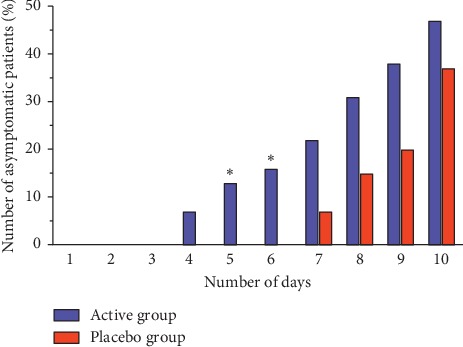
Number of asymptomatic patients in active and placebo groups during the acute treatment phase. ^*∗*^Significant difference (*P* < 0.05) in the number of patients compared to the placebo group. Data represent mean values.

The incidence of the side effects was without significant differences between the groups (*P* = 0.283). On days 5 and 6, we observed a significantly higher number of asymptomatic patients in the active group compared to the placebo group (13% vs. 0%; *P* = 0.027 and 16% vs. 0%; *P* = 0.013, respectively) (Figure 2). There were no significant between-group differences found in the intensity of all herpes symptoms, in the size of the herpes lesions at baseline (*P* = 0.441), and in the absolute change of herpes size within 10 days of treatment (*P* = 0.496).

In all, 63% of patients in the active group and 78% of patients in the placebo group (*P*=0.127) used some form of antiherpetic concomitant therapy during the acute phase. Despite that the duration of herpes was shorter in the active group. Other therapy, nonrelated to the herpes infections, was used by 13.0% and 12.2% of subjects in the active and placebo groups, respectively (*P*=0.905).

**Figure 3:**
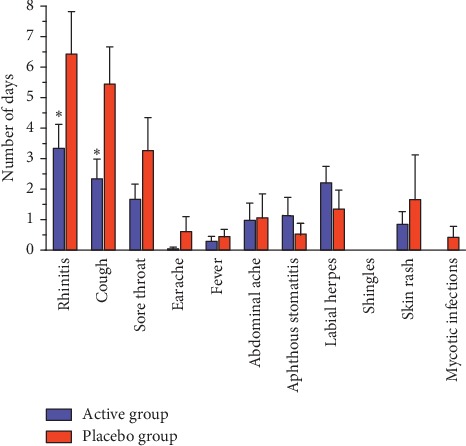
Average duration of the acute respiratory symptoms and intercurrent diseases in active and placebo groups during the preventive phase. ^*∗*^Significant difference (*P* < 0.05) in the number of days compared to the placebo group. Data represent mean ± SEM.

**Figure 4:**
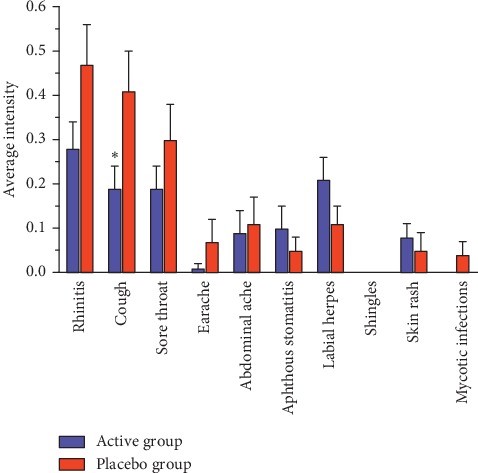
Intensity of the acute respiratory symptoms and intercurrent diseases in active and placebo groups during the preventive phase. ^*∗*^Significant difference (*P* < 0.05) in average intensity of symptoms compared to the placebo group. Data represent mean ± SEM.

The acute treatment phase was followed by a 120-day preventive phase (39 patients in the active group and 38 patients in the placebo group) (Table 1). The sex and age distributions remained similar to the preventive phase. No differences in the prevalence of side effects were observed between the groups (5.1% vs. 5.3%; *P*=1.00). In all, 46.2% of patients in the active group and 63.2% of patients in the placebo group (*P*=0.134) used some form of concomitant therapy (e.g., antibiotics, symptomatic treatment of respiratory infections, and antiherpetic therapy) during the preventive phase. Additionally, we observed a tendency toward lower antibiotic use in the active group (12.8% vs. 28.9%; *P*=0.081). Analysing the general incidence of various acute respiratory symptoms (rhinitis, cough, sore throat, earache, and fever) and intercurrent diseases (abdominal pain, aphthous stomatitis, labial herpes, shingles, skin rash, and mycotic infections), no significant differences between the groups were found. However, detailed analysis revealed some protective and preventive effects of the applied therapy. The duration of rhinitis and cough during the preventive phase was significantly shorter in the active group compared to the placebo group (3.36 ± 0.76 days vs. 6.45 ± 1.36 days; *P*=0.050 and 2.36 ± 0.62 days vs. 5.47 ± 1.19 days; *P*=0.024, respectively) (Figure 3). The intensity of various symptoms generally tended to decrease during the preventive phase; however, a significant difference was found only in the case of cough (0.19 ± 0.05 days vs. 0.41 ± 0.09 days; *P*=0.035) (Figure 4).

## 4. Discussion

This is the first clinical trial studying the potential treatment and preventive effects of a biologically active polysaccharide, pleuran (insoluble *β*-glucan isolated from *Pleurotus ostreatus*), in the management of herpes simplex infections. From 90 patients primarily included in the study, 77 subjects were able to comply with the protocol and finish the study. At the beginning, no differences regarding various characteristics of herpes infection (symptoms and stages of current disease at screening visit and number of labial or facial HSV episodes within the previous 6 to 12 months) were observed between the active and placebo groups. On the other hand, one of the study limitations is related to the different average age in each group (Table 1) which is evident from the fact that the active group included three patients aged over 60 years.

Active treatment with pleuran in systemic application caused shorter duration of herpes simplex symptoms compared to the placebo group. A higher proportion of asymptomatic patients was observed within 5 days in the active group. During the preventive phase, the duration and severity of respiratory symptoms were lower in the active group compared to the placebo group. No significant side effects were observed during both phases of the clinical trial (acute and preventive).

Infections caused by HSV-1 represent a common problem in childhood as well as in adulthood with variable prevalence. Several studies have shown that the seroprevalence against HSV-1 reaches approximately 40% by the age of 15 with a subsequent gradual increase to 60–90% among older adults [29]. Orolabial symptoms caused by HSV-1 are observed in daily clinical praxis more frequently compared to genital herpetic infections. It can occur as a single event, but in certain subjects, it may become recurrent and periodic. The frequency and severity of each episode varies inter- and intraindividually. For the patients with frequent symptoms of orolabial herpes, it can be associated with painful sensations and social stigmatization with decreased quality of life. It has been shown that orolabial herpes simplex belongs to the most stigmatizing dermatologic diseases [30]. Several factors (e.g., emotional stress, physical effort, respiratory tract infections, mild local trauma, exposure to sun light, and menstruation) have been identified as possible triggers for recurrent herpetic symptoms. Moreover, immunodepression/immunosuppression can be the underlying cause associated with all of the abovementioned factors [31]. Clinical management should be rational and should be aimed not only on the treatment of acute symptoms but also on the prevention of recurrent symptoms in the future. Treatment strategies should be aimed at the reduction of the ongoing asymptomatic shedding of the virus, which occurs in the oral mucosa about 20–25% of days of the year [32]. Several antiviral agents were studied in clinical trials (e.g., acyclovir and valacyclovir) in episodic or long-term application. According to the Cochrane Systematic Review, the long-term use of oral antiviral agents can prevent the reoccurrence of labial herpes but the clinical benefit is less [33]. Taking into account the possible undergoing immunodepression, various immunomodulating agents could be another possible option for acute and long-term prophylactic treatment. Several synthetic and natural immunomodulators with possible antiviral activities were studied in the treatment and prevention of labial herpes simplex with various efficacies, including inosine pranobex [34], transfer factors [35–38], topical honey [39], lysine [40], vitamin C [41], and zinc [42].

Biologically active polysaccharides, including *β*-glucans, represent one of the most studied group of natural substances with many beneficial biological activities useful in the management and prevention of various pathological conditions of infectious and noninfectious origin. They possess numerous immunomodulatory, anti-infective, antioxidative, and anti-inflammatory properties, which have been described in *in vitro* murine models as well as human studies [43–45]. Moreover, the revealed mode of action of targeting selected receptors on immune cells put this group of natural substances at the centre of attention of current medicine [21, 22, 46]. However, *β*-glucans from various sources could display different biological effects [47]. In our study, we confirmed the positive therapeutic effect of pleuran on the duration and severity of labial HSV-1 infection. To our knowledge, this is the first human study confirming the beneficial effect of *β*-glucans in the management of labial herpes symptoms. On the other hand, several *in vitro* and animal studies have shown the anti-HSV-1 efficacy of various *β*-glucans and biologically active polysaccharides. In 2011, Santoyo et al. [11] studied the possible antiviral activities of *Boletus edulis, P. ostreatus*, and *Lentinus edodes* extracts against HSV-1 *in vitro.* Their antiviral activity was strong and correlated with the *β*-glucan contents presented in the polysaccharide fractions from the natural sources of active substances. Another source of *β*-glucans, *Ganoderma lucidum,* was also studied. Several neutral and acidic protein-bound polysaccharides showed inhibitory activities against both HSV-1 and HSV-2 [48]. Oat *β*-glucan feeding in mice resulted in a dose-dependent increase of proinflammatory cytokines (IL-1*β*, IL-6, and TNF-*α*) with decreased risk for HSV-1 infection. These cytokines were shown to be important in the physiological immune response and protection against HSV-1 [27]. Oral application of the sulphated derivative of *Agaricus brasiliensis* mycelial polysaccharide also exhibited anti-HSV-1 activity in a murine model. This natural substance reduced disease scores and accelerated the healing of lesions [49].

Several mechanisms could explain the observed anti-HSV-1 effects from murine models and from the findings in the present study. Among the most important modes of action, the stimulation of cytokine production, activation of NK cells and T lymphocytes, and increased production of nitric oxide should be pointed out [50–56]. Another important mechanism could be the activation of DCs, allowing more effective antigen processing and presentation under the condition of HSV-1 infection [57].

During the preventive phase, an additional positive effect on the duration and severity of various respiratory symptoms was also confirmed. The preventive effect of pleuran on respiratory tract infections was confirmed in several open-label studies in children [58–61]. In a double-blind, placebo-controlled clinical trial, pleuran showed preventive effect with respect to respiratory morbidity and exhibited several modulatory effects on humoral immunity and cellular immunity [62]. Its positive effect on respiratory symptoms and infections was confirmed also in patients with Crohn's disease [63] and elite athletes [64]. In a recent systematic review, we evaluated the effect of various *β*-glucans on the treatment and prevention of respiratory tract infections and symptoms [65]. The positive effect of pleuran on respiratory symptoms, their duration, and severity could be explained by its pluripotent biological activities. It supports the production of antibodies, modulates specific cellular immunity, and has positive effect on the activity of NK cells [62, 64]. It also yielded an anti-infectious effect on selected pathogens [28].

Recurrent labial HSV-1 infections are very common in daily practice and represent one of the most stigmatizing dermatological conditions. The use of various natural substances with revealed mode of action is highly required in current clinical practice. According to our study, the use of pleuran seems to be a promising approach in the treatment of acute labial herpes simplex with an excellent safety profile and beneficial effect on the respiratory tract symptoms and infections, which can be one of the possible triggering factors for labial HSV-1 reoccurrence.

## Figures and Tables

**Table 1:** Demographic characteristics of the study groups.

Parameter	Group		*P*
	Active	Placebo	
Acute treatment phase	90 subjects		
Patients (*n*)	49	41	n.s.
Age (years)	25.3 ± 2.3	17.4 ± 1.5	0.005
Males (*n*/%)	17 (34.7)	20 (48.8)	n.s.
Females (*n*/%)	32 (65.3)	21 (51.2)	
Number of herpes exacerbations during 6 months before study (*n*)	2.7 ± 0.5	1.7 ± 0.3	n.s.
Number of herpes exacerbations during 12 months before study (*n*)	4.0 ± 0.5	3.2 ± 0.4	n.s.

Preventive phase	77 subjects		
Patients (*n*)	39	38	n.s.
Age (years)	26.1 ± 2.5	17.6 ± 1.4	0.008
Males (*n*/%)	14 (35.9)	18 (47.4)	n.s.
Females (*n*/%)	25 (64.1)	20 (52.6)	

## Data Availability

The data used to support the findings of this study are available from the corresponding author upon request.
